# Sex-specific association between asthma and hypertension in nationally representative young Korean adults

**DOI:** 10.1038/s41598-017-15722-w

**Published:** 2017-11-15

**Authors:** Hong Seok Lee, Yong-Moon Park, Kyungdo Han, Gerald Pekler, Seong-Su Lee, Soonjib Yoo, Sung Rae Kim

**Affiliations:** 1 Department of Medicine, Metropolitan Hospital Center, New York Medical College, New York, USA; 2 Division of Cardiology, Department of Medicine, St. Bernadine Medical Center, University of California Riverside, California, USA; 3 Epidemiology Branch, National Institute of Environmental Health Sciences, National Institutes of Health, Research Triangle Park, Durham, NC USA; 4 Department of Biostatistics, College of Medicine, The Catholic University of Korea, Seoul, South Korea; 5 Division of Cardiology, Department of Medicine, Metropolitan Hospital Center, New York Medical College, New York, USA; 6 Division of Endocrinology and Metabolism, Department of Internal Medicine, Bucheon St. Mary’s Hospital, College of Medicine, The Catholic University of Korea, Bucheon-si, Gyeonggi-do South Korea

## Abstract

It has been reported that people with asthma have an increased risk of hypertension. However, little is known about the specific relationship between asthma and hypertension in young adults. Among subjects who participated in the Korea National Health and Nutrition Examination Survey conducted in 2008–2013, a total of 10,138 young adults (4,226 men and 5,912 women) aged 19–39 years were analyzed. Multiple logistic regression analysis was used to estimate adjusted odds ratios (ORs) and 95% confidence intervals (CIs). The prevalence of ever asthma was 11.1% in men and 8.4% in women. The mean diastolic blood pressure (DBP) was lower in men with asthma than in men without asthma (*p* = 0.03), whereas the mean DBP was higher in women with asthma than in women without asthma (*p* = 0.04). Having asthma was inversely associated with hypertension in men (OR: 0.62, 95% CI: 0.41–0.91). In contrast, having asthma was positively associated with hypertension in women (OR: 2.19, 95% CI: 1.19–4.02). Our results suggest that asthma pathophysiology might be differentially associated with hypertension in young adults depending on sex.

## Introduction

Asthma is a public health burden in the United States^1^ and in many Asian countries^1,2^. The prevalence has increased from 3.1% in 1980 to 8.4% in 2010 in the United States^1^.

Asthma is a multifactorial disease characterized by airway inflammation, smooth muscle cell proliferation-induced airway remodeling, and smooth muscle contraction^3^. It is often associated with various comorbidities such as rhinitis, sinusitis, gastroesophageal reflux disease, obesity^4^, obstructive sleep apnea^5,6^, and hypertension^7^. A large population-based study found that individuals with asthma were more likely to have hypertension than individuals without asthma, independent of traditional risk factors^7^. Asthma and hypertension may share similar pathophysiology in terms of smooth muscle spastic disorders^8^. Chronic hypoxia due to asthma can increase smooth muscle and connective tissue contents, possibly exacerbating hypertension^9^. In addition, asthma accompanies systemic inflammation with an increased level of interleukin-6 that is related to risk of hypertension^10^.

The prevalence of asthma has been reported to be different depending on sex, owing to various factors. Potential explanations for sex differences in asthma include genetic predisposition related to susceptibility to DNA methylation, differences in immune response such as interleukin levels, hormonal influences, and environmental factors such as differential response to exposures^11^. In contrast, the prevalence of hypertension is also different between men and women^12^. It has also been reported that asthma is associated with increased cardiovascular risk, especially in women^13^.

A number of epidemiological studies have explored the prevalence of hypertension in patients with asthma with different levels of severity or its prevalence between individuals with and without asthma^14^, especially in older adults^12^. However, few studies have investigated the association between asthma and hypertension in young adults. In addition, previous studies had several limitations, including localized study areas^15^, or little consideration of potential confounders such as allergic rhinitis^16^, obesity^17^, stress level^18,19^, sleep duration^20^, body mass index (BMI)^21^, or vitamin D status^22^, as well as a potential co-morbidity for hypertension such as allergic respiratory disease^16^. Furthermore, little is known about the relationship between asthma and hypertension in Asian countries. Therefore, the objective of this study was to determine the relationship between asthma and hypertension in young adults depending on sex by using a nationally representative sample.

## Methods

### Study population

For this study, the nationally representative cross-sectional Korea National Health and Nutrition Examination Survey (KNHANES) was used. It was a survey of the non-institutionalized Korean civilian population conducted by the Korea Centers for Disease Control and Prevention. Sampling units were defined based on characteristics such as age, sex, and geographical region collected from household units in the 2005 National Census Registry. To produce an unbiased national estimate, sample weights were assigned for participating individuals reflecting unequal probability of selection with nonresponse and population structure adjustment to represent the Korean population. The KNHANES comprised three parts: a health interview survey, a health examination survey, and a nutrition survey. Among subjects who participated in the 2008–2013 KNHANES, a total of 10,138 young adults (4,226 men and 5,912 women) aged <40 years were analyzed. In Korea, all citizens who are 40 years or older are eligible for receiving national health insurance-covered health screening tests including that for hypertension^23^. Therefore, the age of 40 years was chosen as the upper age limit for young adults in this study, given that individuals aged ≥ 40 years are more likely to be diagnosed with hypertension compared to those aged <40 years. This could result in a differential selection of outcomes in this population according to age.

### Measurements

All subjects’ variables were measured by trained examiners. The KNHANES health interview information survey collected respondents’ demographic status and health-related characteristics (including age, education, household income, and the use of antihypertensive medication). In addition, self-administered questionnaires included lifestyle characteristics such as smoking, drinking alcohol, and exercise. High school level of education (12 years) is mandatory in Korea. Therefore, education was dichotomized to two groups depending on whether college level of education was obtained (i.e., ≤12 years and >12 years). In terms of income, monthly salary, real estate, pension, interest rate, and any support from government were surveyed and combined as total household income. Income quartiles were determined by 5-year age groups and survey year for each sex and used as a summary value. The median (range) values of the cutoffs for income quartiles were 100 (55, 125), 150 (106, 192) and 214 (173, 250) in men, and 100 (71, 139), 150 (106, 200) and 224 (191, 283) in women, respectively (the monetary unit is 10,000 won, which is equivalent to approximately 9 US dollars).

The smoking status included current smokers, past smokers, and non-smokers. The survey categorized alcohol consumption based on the average frequency and amount of alcoholic beverage that were consumed. These were converted into the amount of pure alcohol (in grams) consumed per day and categorized into the following three groups: none, mild to moderate drinker (<30.0 g alcohol/day), and heavy drinker (≥ 30.0 g alcohol/day). The survey categorized regular exercise as either strenuous (more than 30 min at a time, more than five times a week) or moderate (more than 20 min at a time, more than three times per week). The amount of exercise was measured based on its intensity. No activity was considered if subjects did not exercise regularly. Regular exercise was categorized into moderate and vigorous exercise. Vigorous aerobic exercise included running (such as jogging), climbing, cycling, fast swimming, tennis, soccer, basketball, jump rope, and squash. Moderate exercise included slow swimming, table tennis, badminton, and light weightlifting. Regular exercise also included vigorous exercise (more than 30 min at a time, more than five times a week) and moderate exercise (more than 20 min at a time, more than three times per week). No exercise was defined when participants did not perform any physical activity in their leisure-time. The KNHANES used ASSET^24^ to assess whether respondents regularly perceived stress. They were defined as being depressed if they reported more than two weeks of sadness that interfered with daily life in the past year. Perceived stress has been determined in an epidemiological study to be a risk factor for asthma^18^. Moreover, it has been shown that hypertension combined with asthma has a significant relationship with mental distress. In addition, it has been reported that perceived stress can increase the inflammatory process, resulting in hypertension^25^. Therefore, perceived stress was regarded as a risk factor for the assessment^25^.

The survey calculated each respondent’s BMI as an individual’s weight (in kilograms) divided by the square of the individual’s height (in meters). According to the Regional Office for the Western Pacific (WPRO) standard, obesity was considered when BMI was greater than 25. Asians have different criteria for obesity^26^, given that associations between BMI and health risks are different between Asian and western populations^27^. Waist circumference was measured to the nearest 0.1 cm in a horizontal plane at the midpoint between the iliac crest and the costal margin at the end of a normal expiration^28^. Blood samples were obtained in the morning following an overnight fast for at least eight hours. Respondents’ serum concentrations of glucose, high-density lipoprotein cholesterol, and triglycerides were measured using a Hitachi automatic analyzer 7600 (Tokyo, Japan).

### Definition of asthma and hypertension

For our study purpose, asthma was defined as individual self-report of ever receiving a diagnosis of asthma or if subjects reported that they had asthma *plus* chest wheezing/whistling in the past. Patients who did not mention any symptoms of asthma or did not have prior diagnosis of asthma at the time of the study were excluded. In addition, further questions (shown as follows) were used to categorize asthma: (1) For the last one year, have you experienced wheezing or whistling? (2) Have you ever used any asthma medication? (3) Have you ever been to an emergency room due to asthma exacerbation? (4) Have you ever experienced aggravating symptoms of asthma? In our study, the diagnosis was based on previous medical history or personal report, therefore, overdiagnosis or misclassification could be included. Blood pressure (BP) was measured three times on the right arm using a mercury sphygmomanometer (Baumanometer; Baum, Copiague, NY, USA) while the individual was at rest in a seated position for at least 5 minutes. Final BP was obtained by averaging the second and third BP measurements following the international guideline^29^. A minimum of two readings were taken at intervals of at least a few minutes. Then, the average of these readings was used to represent the patient’s blood pressure. Our study used the Seventh Report of the Joint National Committee (JNC 7) on High Blood Pressure’s^30^ definition. Prehypertension was considered when systolic BP (SBP) was 120–139 mmHg or diastolic BP (DBP) was 80–90 mmHg. The JNC 7 defined hypertension as SBP ≥ 140 mmHg and DBP ≥ 90 mmHg. For patients with diabetes, the targeted BP was below 130/80 mmHg^30^. The National Cholesterol Education Program Adult Treatment Panel III blood pressure criterion for metabolic syndrome is SBP over 130 mmHg and DBP over 85 mmHg^31^. Hypertension was defined in participants if they were taking antihypertensive medication.

### Statistical analysis

All statistical analyses were performed using SAS Version 9.3 (SAS Institute, Cary, NC, USA) to account for the complex sampling design and provide nationally representative prevalence estimates. For subgroup analysis, domain option was applied to preserve appropriate subsamples in the complex sampling design. Subjects’ characteristics were presented as mean with standard error (SE) for continuous variables or percentage with SE for categorical variables. Linear regression analysis was used to compare continuous variables. Rao-Scott chi-square test was used to compare proportions. Proportions of subjects’ asthma were ascertained for selected socioeconomic, lifestyle, and comorbidity factors.

It is known that there is a sex-specific association between asthma and metabolic health outcomes^32^. Therefore, all analyses by sex were performed separately. Multiple logistic regression analysis was performed to test the association between asthma and hypertension after adjusting for potential confounders identified *a priori* based on literature review. Men and women with asthma had common risk factors such as BMI, smoking history, and drinking alcohol, all of which are associated with hypertension. However, sleep duration was a significant risk factor for men with asthma. In addition to the crude model, age was adjusted for in model 2. In model 3, BMI^28^, smoking (non-smoking, current smoking, ex-smoker)^33^, drinking alcohol (non-drinker, mild to moderate drinker, and heavy drinker)^34^, regular exercise (regular, non-regular, no exercise)^35^, income (quartiles)^36^, perceived stress^37^, and sleep duration^20^ were adjusted for. In model 4, serum vitamin D concentration^22^ and other allergic diseases (atopic dermatitis, allergic rhinitis) were adjusted for. Statistical significance was determined with two-sided tests. Significance level α was set at 0.05.

## Results

Differences in general characteristics by sex are summarized in Table 1. In our study, men and women who had been diagnosed with asthma were relatively younger (*p* = 0.03 and *p* = 0.04, respectively) compared to those without asthma. They were also more likely to be current smokers and heavy drinkers compared to individuals without asthma (all *p* < 0.05). Patients of both sexes who had asthma also had prior diagnoses of atopic dermatitis and allergic rhinitis (all *p* < 0.01). Both men and women with asthma were more likely to report psychological factors such as stress and depression (all *p* < 0.01). Since this study was based on an apparently healthy general population, clinical information such as asthma medication, age of diagnosis, or asthma severity of control was not included.

**Table 1:** General characteristics of the study population according to the presence or absence of asthma by sex (N = 10,138).

	Men			Women		
	Without Asthma	With Asthma	P-value	Without Asthma	With Asthma	P-value
Variables	(n = 3775)	(n = 451)		(n = 5453)	(n = 459)	
**Age (years)**	29.7 ± 0.1	29.1 ± 0.3	0.03	29.8 ± 0.1	29.0 ± 0.4	0.04
**Smoker**			<0.01			<0.01
Non	34.3(1)	22.2(2.3)		87.2(0.6)	74.2(2.4)	
Ex-smoker	12.5(0.6)	9.4(1.7)		5.1(0.4)	6.4(1.3)	
Current	53.2(1)	68.4(2.6)		7.8(0.5)	19.4(2.1)	
**Alcohol Drinking** (%)			<0.01			0.02
Non	7.5(0.5)	8.4(1.6)		20.5(0.7)	14.5(1.8)	
Mild to Moderate	75.9(0.8)	65.5(1.2)		75.6(0.7)	78.9(2.2)	
Heavy	16.7(0.7)	27.5(2.5)		3.9(0.4)	6.6(1.3)	
**Exercise** (%)			0.02			0.03
Regular	16.0(0.7)	22.0(2.3)		7.0(0.4)	10.8(1.8)	
Non-regular	66.4(0.9)	60.8(2.6)		79.2(0.7)	77.4(2.4)	
No exercise	17.6(0.7)	17.2(2.0)		13.8(0.6)	11.8(1.6)	
**Income**			0.16			0.05
Lowest tertile	9.4(0.7)	6.6(1.4)		7.6(0.5)	11.9(2)	
Middle tertile	59.7(0.9)	64.5(2.4)		61.7(0.8)	60.1(2.4)	
Highest tertile	30.8(1)	30(2.5)		30.7(0.9)	28(2.5)	
**Education (≥12 years)**	46.1(1)	34.2(2.5)	<0.01	49(0.9)	45.8(2.9)	0.26
**Atopic Dermatitis (%)**	4.2(0.4)	8.5(1.6)	<0.01	5.2(0.4)	10.8(1.8)	<0.01
**Allergic Rhinitis (%)**	18.7(1.3)	29.8(4.5)	0.01	21.7(1.2)	37.4(4.4)	<0.01
**Asthma diagnosed by physician**	0(0)	24.6(2.3)	.	0(0)	27.8(2.4)	.
**Stress (%)**	28.8(0.8)	43.9(2.6)	<0.01	35.6(0.8)	46.9(2.7)	<0.01
**Depression (%)**	7.6(0.5)	12.7(1.9)	<0.01	14.5(0.6)	20.9(2.2)	<0.01
**Mean sleep hours**	6.9 ± 0	6.8 ± 0.1	0.78	7.2 ± 0	7.2 ± 0.1	0.07
**Serum Vitamin D concentration**	17.3 ± 0.2	17.1 ± 0.3	0.65	15.4 ± 0.1	15.3 ± 0.3	0.79

Data are shown as means ± standard error (SE) or percentages (SE).

Abbreviations: SBP; systolic blood pressure, DBP; diastolic blood pressure; Depression was defined if participants felt sadness or agony for over two weeks that was interfering with daily life for the last one year; heavy smoking: at least one pack a day.

Results of the comparison of general characteristics of participants with hypertension, prehypertension, and normotension by sex are shown in Table 2. Alcohol intake, income level, education, and asthma diagnosed by a physician were significantly different (*p* < 0.05) among the abovementioned three groups in men. In women, age, systolic blood pressure, diastolic blood pressure, anti-hypertensive, smoking, income level, education, and psychological stress were significantly different (*p* < 0.05) among the three groups. The prevalence of hypertension in our study population was 14.1% in men and 2.5% in women. The prevalence of ever asthma was 11.1% in men and 8.4% in women.

**Table 2:** Comparison of general characteristics among hypertension, prehypertension, and normotension groups by sex.

	Men				Women			
	Normotension	Prehypertension	Hypertension	P-value	Normotension	Prehypertension	Hypertension	P-value
Variables	(n = 2131)	(n = 1481)	(n = 614)		(n = 4939)	(n = 764)	(n = 170)	
**Age (years)**	29.03 ± 0.2	29.9 ± 0.2	31.4 ± 0.3	<0.01	29.5 ± 0.1	30.6 ± 0.3	33.4 ± 0.4	<0.01
**Smoking**				0.36				0.05
Non	86.4(0.6)	85.2(1.5)	80.4(3.8)		33.3(1.2)	33.7(1.5)	29.4(2.1)	
Ex-smoker	5.1(0.4)	5.8(1.1)	6.1(2.1)		11.2(0.8)	12(0.9)	16.1(1.7)	
Current	8.6(0.5)	9(1.3)	13.5(3.4)		55.5(1.2)	54.3(1.5)	54.4(2.4)	
**Alcohol Drinking**				<0.01				
Non-drinker	20.3(0.7)	18.9(1.6)	16.9(3.3)		8.2(0.7)	7.4(0.8)	5.8(1.1)	
Mild to moderate-Drinker	76.1(0.8)	74.4(1.9)	75.2(4)		78.8(1)	72.5(1.3)	65.3(2.1)	
Heavy-drinker	3.6(0.4)	6.7(1.2)	7.9(2.6)		13.1(0.9)	20.1(1.2)	28.8(2)	
**Exercise**				0.30				0.75
Regular	7.2(0.5)	8.8(1.3)	5.2(2)		16(0.9)	17.8(1.2)	16.3(1.7)	
Non-regular	79.1(0.7)	78.9(1.8)	77.3(3.7)		66(1.2)	65.4(1.4)	65.9(2.2)	
No exercise	13.7(0.6)	12.3(1.3)	17.5(3.2)		18(1)	16.8(1.1)	17.8(1.7)	
**Income**				0.01				0.01
Lowest quartile	7.8(0.6)	7.1(1)	15.3(3.2)		9.8(0.9)	8(0.8)	9.5(1.5)	
Middle quartile	61.2(1)	64(2.1)	60.9(4.1)		59.4(1.3)	59.7(1.6)	64(2.2)	
Highest quartile	31(0.9)	28.9(1.9)	23.7(3.4)		30.8(1.3)	32.3(1.6)	26.5(2)	
**Atopic dermatitis (%)**	5.5(0.4)	6.3(1)	6.1(2.1)	0.77	4.6(0.5)	4.6(0.6)	5.4(1.1)	0.79
**Allergic Rhinitis (%)**	22.9(1.3)	24.5(3)	16.2(5.9)	0.54	21.3(2)	17.7(1.9)	20.2(3)	0.37
**Education ( ≥ 12 years) (%)**	50(0.9)	44.2(2.2)	33.7(4.1)	<0.01	42(1.3)	47.4(1.5)	48.2(2.4)	0.01
**Psychological Stress (%)**	36(0.8)	39.3(2.1)	42(4.4)	0.13	29.2(1.1)	30.1(1.4)	35.8(2.2)	0.02
**Mean sleep hours**	7.2 ± 0.1	7.3 ± 0.1	7.1 ± 0.1	0.64	6.9 ± 0.0	6.9 ± 0.0	6.9 ± 0.1	0.85
**Depression (%)**	14.9(0.6)	15.5(1.6)	15.7(3.4)	0.93	8.5(0.7)	7.7(0.8)	7.9(1.2)	0.71
**Vitamin D (mg/dl)**	17.2 ± 0.3	17.4 ± 0.2	17.4 ± 0.3	0.81	15.4 ± 0.1	15.6 ± 0.3	15.6 ± 0.5	0.68

Data are presented as means ± standard error (SE) or percentages (SE). Abbreviations: SBP, systolic blood pressure, DBP, diastolic blood pressure.

Results of the comparison of metabolic abnormalities between individuals with asthma and those without asthma by sex are shown in Table 3. The mean DBP was significantly lower in men with asthma than in men without asthma (*p* = 0.03). However, the mean DBP was significantly higher in women with asthma than in women without asthma (*p* = 0.04). High BP has been defined as one component of metabolic syndrome^12^. The proportion of high BP was higher in women with asthma than in women without asthma (*p* = 0.01). In contrast, the proportion of high BP was lower in men with asthma than in men without asthma (*p* = 0.04). The proportion of general obesity was higher (*p* = 0.03) in men with asthma than that in men without asthma.

**Table 3:** Comparison of metabolic abnormalities between asthma and non-asthma participants by sex.

	Men			Women		
	Without Asthma	With Asthma	P-value	Without Asthma	With Asthma	P-value
Variables	(n = 3775)	(n = 451)		(n = 5453)	(n = 459)	
SBP (mmHg)	115.5 ± 0.2	114.4 ± 0.6	0.09	104.9 ± 0.2	106.2 ± 0.7	0.08
DBP (mmHg)	77.6 ± 0.2	76.6 ± 0.5	0.03	69.6 ± 0.2	70.9 ± 0.6	0.04
Anti-hypertensive meds (%)	1.0(0.2)	0.9(0.5)	0.89	0.3(0.1)	2.0(0.7)	<0.01
Hypertension (%)	14.1(0.7)	10.5(1.5)	0.05	2.5(0.2)	6.2(1.4)	0.001
BMI(kg/m^2^)	24 ± 0.1	24.3 ± 0.2	0.21	22 ± 0.1	22.3 ± 0.2	0.30
WC (cm)	82.5 ± 0.2	83.4 ± 0.6	0.15	73.7 ± 0.2	74.6 ± 0.6	0.13
Obesity (BMI ≥ 25) (%)	34.1(0.9)	40.2(2.6)	0.03	17.4(0.7)	19.1(2.1)	0.44
High WC* (%)	20(0.8)	24(2.3)	0.08	21.9(0.8)	24.3(2.3)	0.29
High blood pressure† (%)	14.1(0.7)	10.5(1.5)	0.04	2.5(0.2)	6.2(1.4)	0.01
High blood glucose‡ (%)	13.9(0.7)	13.9(2)	0.98	7.3(0.4)	8.9(1.5)	0.26
High triglycerides§ (%)	28.7(0.9)	32.5(2.6)	0.15	9.8(0.5)	11.7(1.9)	0.27
Low HDL-C|| (%)	17.1(0.7)	16.3(2.1)	0.72	25.7(0.8)	24.1(2.2)	0.49
Vitamin D (mg/dL)	17.3 ± 0.2	17.1 ± 0.3	0.63	15.4 ± 01	15.3 ± 0.3	0.03
Metabolic syndrome¶ (%)	13(0.7)	13.8(1.9)	0.71	6.3(0.4)	8.8(1.7)	0.10
Diabetes mellitus (%)	1.9(0.2)	2.6(1)	0.42	1.3(0.2)	2.3(0.8)	0.10

Data are presented as means ± standard error (SE) or percentages (SE).

Abbreviations: SBP, systolic blood pressure; DBP, diastolic blood pressure.

BMI, body mass index; WC, waist circumference; HDL-C, high-density lipoprotein cholesterol; *Waist circumference ≥ 90 cm in men and ≥ 80 cm in women.

^†^Systolic blood pressure/diastolic blood pressure ≥ 130/85 mmHg or use of antihypertensive medication for self-reported hypertension.

^‡^Fasting blood glucose ≥ 100 mg/dL or antidiabetic medication use.

^§^Triglycerides ≥ 150 mg/dL or on cholesterol-lowering medication.

^||^HDL-C < 40 mg/dL in men and < 50 mg/dL in women or on cholesterol-lowering medication.

^¶^Subjects with three or more of the above five metabolic conditions.

**Figure 1:**
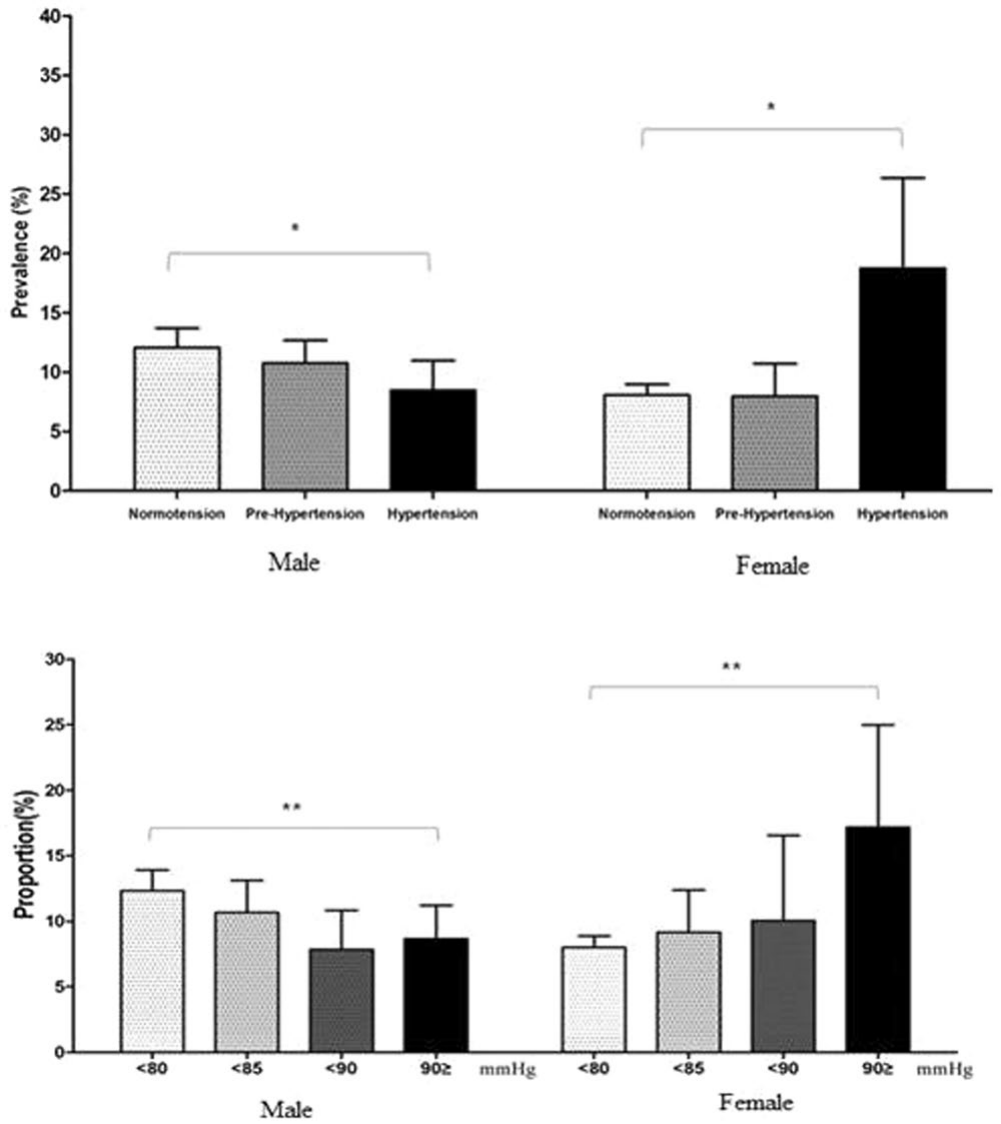
Prevalence of asthma according to normotension, prehypertension, hypertension by sex. Hypertensive men had low prevalence of ‘ever’ asthma (*p* = 0.03) and hypertensive women had high prevalence of ‘ever’ asthma (*p* = 0.02).

The prevalence of ever asthma with respect to hypertension, prehypertension, and normotension is shown in Fig. 1. Men with ever asthma had a low prevalence of hypertension but a high prevalence of normotension. There were significant differences (*p* = 0.03) in the prevalence among the three groups. In contrast, women with ever asthma had a significantly higher (*p* = 0.02) prevalence of hypertension compared to those without ever asthma.

**Figure 2:**
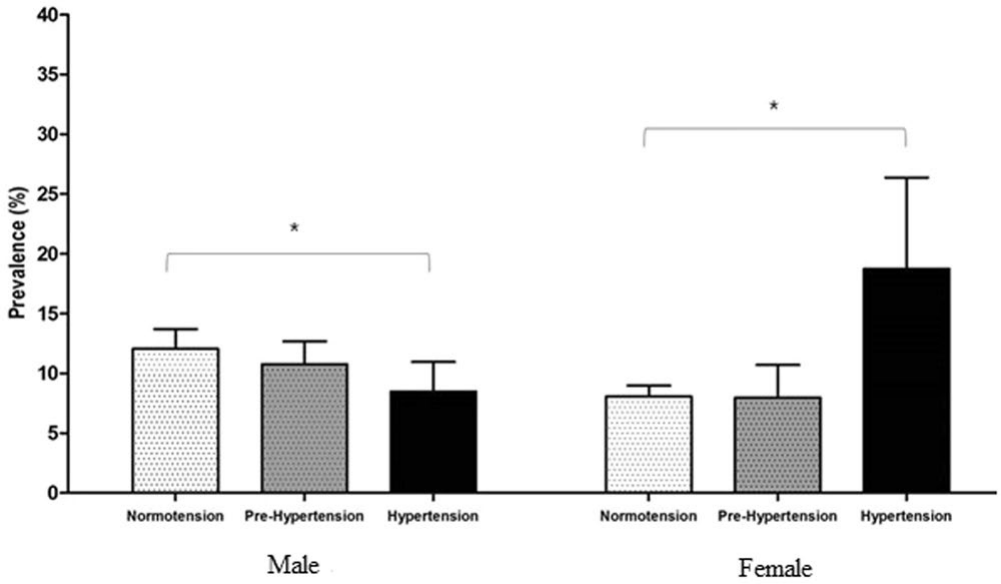
(**A**,**B**) Distribution of asthma with increasing systolic and diastolic blood pressure by sex. With increasing systolic blood pressure, prevalence of ‘ever’asthma tended to decrease in men (*p for trend* = 0.04) and increase in women (*p for trend* < 0.01). Similarly, with increasing diastolic blood pressure, prevalence of ‘ever’asthma tended to decrease in men (*p for trend* < 0.01) and increase in women (*p for trend* < 0.01).

There was an opposite trend in systolic blood pressure between men and women with ever asthma (Fig. 2). Men showed a decreasing prevalence of ever asthma as systolic blood pressure increased (*p* = 0.04). However, women showed an increasing prevalence of ever asthma as systolic blood pressure increased (*p* < 0.01). This pattern was the same for diastolic blood pressure in men (*p* < 0.01) and women.

Men who had been diagnosed with ever asthma were less likely to have hypertension (OR: 0.62, 95% CI: 0.41–0.91) compares to men without ever asthma after adjusting for age, BMI, smoking, drinking alcohol, physical activity, income, perceived stress, sleep duration, serum vitamin D concentration, and coexisting allergic conditions such as atopic dermatitis and allergic rhinitis (Table 4). In contrast, women with ever asthma were more likely to have hypertension compared to women without asthma (OR: 2.19, 95% CI: 1.19–4.02).

**Table 4:** Odds ratios and 95% confidence interval for the association between asthma and hypertension by sex.

	Men	Women
Model 1	0.71 (0.51, 0.99)	2.63 (1.57, 4.41)
Model 2	0.74 (0.53, 1.04)	2.89 (1.71, 4.87)
Model 3	0.67 (0.46, 0.98)	2.25 (1.26, 4.00)
Model 4	0.62 (0.41, 0.91)	2.19 (1.19, 4.02)

Data are presented as odds ratio (95% confidence interval).

Model 1: unadjusted;

Model 2: adjusted for age;

Model 3: adjusted for age, BMI, smoking, drinking alcohol, physical activity, income, perceived stress, and sleep duration;

Model 4: adjusted for covariates used in model 3 as well as coexisting allergic conditions such as atopic dermatitis and allergic rhinitis, and serum vitamin D concentration.

## Discussion

In this nationally representative cross-sectional study using Korean adults aged 19 to 40 years, a positive association between asthma and hypertension in women was found after adjusting for potential confounders. In men, an inverse association between the two conditions was found. To the best of our knowledge, this is the first large and population-based study that investigated the relationship between asthma and hypertension among young adults depending on sex in an Asian population.

Differential association between asthma and hypertension by sex in young adults may suggest sex-dependent pathophysiology of asthma with hypertension. In an *in vivo* study, testosterone had an effect on systemic vasodilatation in male animals, which might suggest that testosterone has an effect on patients with hypertension having asthma^38^. Testosterone was reported as having vasodilator and vasorelaxant effects, which could be associated with the prevention of hypertension^38^. Usually, hormonal changes can influence variations in disease prevalence and airway remodeling^39^. Estrogen levels and certain estrogen forms such as estradiol, estriol, and estrone in women^40^ might interact with the immune response^41^ and inflammatory response at a cellular level^42^. Another possible explanation is that sex-specific male hormones such as testosterone might have protective effects on vascular remodeling, which is associated with chronic local inflammation of the airway, one of the cardinal features of asthma^43^. As a result, these mechanisms might have influenced sex-specific predominance of asthma and different prevalence of hypertension in our study.

Young women with hypertension could have potential complications such as gestational hypertension, preeclampsia, and eclampsia during pregnancy^44^. Such complications can increase the risk of long-term cardiovascular morbidity and mortality^45^. Therefore, it would be meaningful to understand hypertension risk in young adults by sex. As a chronic medical condition, asthma can synergistically contribute to complications of hypertension^46^. It has been reported that individuals with asthma have increased cardiovascular risk, especially in women^47^. In terms of clinical significance, however, little evidence exists for clinically meaningful change in hypertension that could be attributed to asthma prevention or management, especially in young adults. For instance, the JNC 8 did not include specific guidelines for patients with asthma^48^. Further studies are warranted to provide this evidence.

The association between asthma and hypertension could be related to cardiac burden^49^, which can ultimately affect blood pressure. Patients with asthma have higher cardiovascular disease prevalence than the general population. Their hearts may tend to experience more pressure and volume overload as they pump more rapidly in attempting to cycle more blood through the body and return it to the correct oxygen level. Such increased blood pumping rates could increase blood pressure levels and trigger other issues. As there is a lack of information on asthma such as severity, we cannot conclude that asthma definitely affects cardiac burden. However, we can suggest a potential relationship between asthma severity and hypertension.

In the present study, the prevalence of ever asthma was 11.1% in men and 8.4% in women, whereas the prevalence of hypertension was 13.7% in men and 2.8% in women. Among regional studies in the United States, one study has shown that the prevalence of hypertension in patients with asthma is 54.5–57.3%^46^, which is higher than that observed in this study. However, participants of that study had a mean age of approximately 54 years^46^, while our participants consisted of younger people (less than 40 years old). The prevalence of hypertension was low in women in this study, limiting the ability to draw an inference from such result. This could lead to biased association between asthma and hypertension.

Numerous studies have found that the prevalence of hypertension ranges from 9% to 52%^10^. One study used a population of patients who had already been diagnosed with asthma and who were middle-aged or older (over 50 years old)^10^. Therefore, the prevalence of hypertension might have been also affected by other metabolic factors. In addition, approximately 80–90% of that study population had been previously exposed to inhaled corticosteroids which could be related to the development of hypertension^10^. In the present study, asthma was more prevalent in men than in women, which is different from the results of a 2013 report by the U.S. Centers for Disease Control and Prevention^50^. Our study results suggest that the prevalence of asthma in Asian populations might be higher than that among other ethnicities, similar to the finding of another study^51^. This also suggests that asthma in Asians might have been underdiagnosed and undertreated. In addition, different factors might affect asthma and hypertension in these populations.

There are several limitations in the present study. Due to the cross-sectional design, we could not obtain any causal relationship between asthma and hypertension. In addition, clinical information such as asthma medication, age at diagnosis, or asthma severity was not included, which could reduce the validity of our results. Dietary variables were not included as a covariate in the model due to changes in dietary assessments in KNHANES over the study period. Instead, given the evidence on the association between plasma concentrations of 25 hydroxy vitamin D (25(OH)D), hypertension and asthma^52^, plasma concentration of 25(OH)D was used as an additional covariate in this study. Additionally, self-reported asthma without information on validity or reliability might lead to misclassification or overdiagnosis, which could not be addressed in the present study. Previous studies have shown that asthma in children was overdiagnosed, ranging from 41% to 83.9%, suggesting that overdiagnosis could bias our results^53,54^. Low prevalence could be another limitation of this study. In addition, it has been reported that metabolic derangement could be more prevalent in young and middle-aged adults than that in old-aged adults^12^. Therefore, it is important to consider the cardiovascular risk factors, including hypertension, in younger people in future studies.

However, we used a nationally representative population of young adults who did not have risk factors related to hypertension compared to older respondents with more comorbidities. This may have contributed to reducing the influence of other comorbidities on the association between asthma and hypertension. A strength in the present study is that we considered multiple potential confounders such as obesity^28^, metabolic syndrome^55^, socioeconomic status^56^, psychiatric problems such as stress and depression^37^, and lifestyle variations including smoking and drinking^33^ in the association between asthma and hypertension.

In conclusion, there was a positive association between asthma and hypertension in young women and an inverse association between asthma and hypertension in young men after adjusting for potential confounders. Our study results suggest that asthma pathophysiology could be differentially associated with hypertension in young adults by sex. Thus, sex-specific management of hypertension may be needed for young patients with asthma. Our findings need to be confirmed in prospective cohort and intervention studies in the future.
